# Mobile Network Performance and Technical Feasibility of LTE-Powered Unmanned Aerial Vehicle

**DOI:** 10.3390/s21082848

**Published:** 2021-04-18

**Authors:** Muhammad Aidiel Zulkifley, Mehran Behjati, Rosdiadee Nordin, Mohamad Shanudin Zakaria

**Affiliations:** 1 Department of Electrical, Electronics and Systems Engineering, Faculty of Engineering and Built Environment, Universiti Kebangsaan Malaysia, Bangi 43600, Selangor, Malaysia; aidiel.zulkifley@gmail.com (M.A.Z.); mehran.behjati@ukm.edu.my (M.B.); 2 Faculty of Information Science and Technology, Universiti Kebangsaan Malaysia, Bangi 43600, Selangor, Malaysia; msz@ukm.edu.my

**Keywords:** UAV, drone, BVLoS, wireless, cellular, 4G, LTE

## Abstract

Conventional and license-free radio-controlled drone activities are limited to a line-of-sight (LoS) operational range. One of the alternatives to operate the drones beyond the visual line-of-sight (BVLoS) range is replacing the drone wireless communications system from the conventional industrial, scientific, and medical (ISM) radio band to a licensed cellular-connected system. The Long Term Evolution (LTE) technology that has been established for the terrestrial area allows command-and-control and payload communications between drone and ground station in real-time. However, with increasing height above the ground, the radio environment changes, and utilizing terrestrial cellular networks for drone communications may face new challenges. In this regard, this paper aims to develop an LTE-based control system prototype for low altitude small drones and investigate the feasibility and performance of drone cellular connectivity at different altitudes with measuring parameters such as latency, handover, and signal strength. The measurement results have shown that by increasing flight height from ground to 170 m the received signal power and the signal quality levels were reduced by 20 dBm and 10 dB respectively, the downlink data rate decreased to 70%, and latency increased up to 94 ms. It is concluded that although the existing LTE network can provide a minimum requirement for drone cellular connectivity, further improvements are still needed to enhance aerial coverage, eliminate interference, and reduce network latency.

## 1. Introduction

Unmanned aerial vehicles (UAVs), also known as drones, are becoming increasingly used in a wide variety of cases such as inspection, surveillance, package delivery, medical delivery, and agriculture [1,2,3,4,5,6,7,8]. To ensure safe operation, drones need a secure and stable wireless connectivity for control and command (CC) and payload communications [9]. Conventionally, drones operate on the licensed-free industrial, scientific, and medical (ISM) radio band (2.4 GHz) which the operational range of drones is limited to the visual line-of-sight (LoS) range [10]. To unleash drones’ potential, a beyond visual line-of-sight (BVLoS) connectivity is required to be established [11].

The cellular network has recently been considered one of the main enablers for developing advanced drone use cases [12]. Cellular networks can provide wide-area, quality of service (QoS), high data rate, low latency, and reliable connectivity for both terrestrial [13] and drone user equipments (UEs) [14]. In addition, utilizing the existing terrestrial cellular networks for drone connectivity can facilitate the drone ecosystem’s growth, without the need to develop a new dedicated infrastructure for drone wireless communications [15]. However, using existing cellular networks to provide connectivity to the flying drone seems challenging. With an increasing height above the ground the radio environment changes [15], and some problems arise, such as mobility management [16] and severe interference between drone UEs and terrestrial UEs [15].

**Figure 1:**
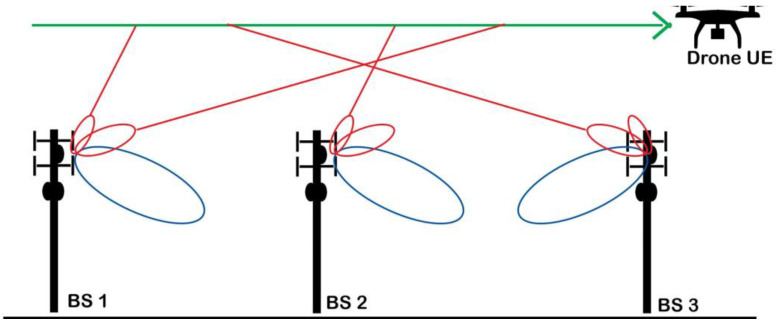
Illustration of wide-area wireless connectivity for a low-altitude drone with terrestrial cellular networks.

Figure 1 illustrates a scenario when a low altitude small drone flies above the terrestrial base stations (BSs). Due to the high probability of line-of-sight (LoS) link in higher altitudes, drone communications suffer from both uplink and downlink interferences [17]. Moreover, terrestrial network planning is optimized for ground UEs, and BS antennas are tilted downwards to prevent inter-cell interference and provide service for ground UEs via antennas’ main lobes [18]. Meanwhile, drones that fly above the BSs are served by the antennas’ sidelobes, which cause severe challenges for interference and mobility management [11].

To better understand the Long Term Evolution (LTE) network’s capability to provide wireless connectivity for low altitude small drones, the 3rd Generation Partnership Project (3GPP) conducted research on enhanced LTE support for aerial vehicles [19]. The primary purpose of that study was to evaluate drone UEs’ mobility performance, including the handover (HO) procedure and cell selection. In addition, recently theoretical research has been conducted on topics such as autonomous navigation [20], joint trajectory and communication design [21], energy efficiency and trajectory optimization [22,23], hybrid-duplex UAV with join detection [24], 3D coverage analysis [25], and spectrum sharing [26]. Among these research topics, channel measurement and modeling have drawn academic interest because of the behavior changes of radio channels at high altitude and their importance in designing reliable communication links for the drones’ safety control and operations.

The works described in [14,15,17,27] studied the radio environment changes with altitude and mobility performance of cellular-connected drones based on simulation results. These works investigated drone connectivity by considering parameters such as signal to interference plus noise ratio (SINR), reference signal received power (*RSRP*), and HO under different drone speeds and heights. The results show that by increasing the drone height the probability of LoS communications links increases, which results in higher interference in neighbor cells compared to terrestrial UEs. Moreover, results show how drones are served by sidelobes of BSs’ antennas and reveal problems of handover when drones move to the BS antenna sidelobe nulls. However, these works are limited to simulation results and do not fully reflect the challenges of real-world scenarios.

The works described in [16,28,29] investigated the performance of drone cellular connectivity by performing field measurements. The articles analyzed the performance of drone connectivity in LTE/LTE-Advanced networks in terms of coverage, data-rate, interference, and latency. Although results show the capability of 4G networks for providing communication links for low altitude drones, little work has yet been done to investigate the impact of HO, latency, coverage, and data rate of cellular-connected drones.

In this regard, the goal of this article is to further shed light on the feasibility and challenges of utilizing the LTE network for providing BVLoS connectivity for drones. To this end, first an LTE-based cellular-connected drone prototype was developed for conducting field measurement. Then, a field trial measurement was performed to investigate the performance of drone connectivity at different altitudes in terms of *RSRP*, reference signal received quality (*RSRQ*), data-rate, latency, and HO.

The key contributions of this paper are summarized as follows:

- An LTE-powered drone prototype was constructed based on the latest hardware and software available in the market. The developed drone prototype can handle less delayed video streaming, send a real-time location, and be fully controlled using the LTE mobile network remotely. The prototype design can guide future works in this area.
- A field trial measurement was conducted in a commercial LTE network and suburban environment (university campus). For measurements, the state-of-the-art techniques were used and important parameters, such as *RSRP*, *RSRQ*, data rate, latency, and handover, were measured to evaluate the system performance.
- Performance of the LTE system was analyzed according to the measured parameters and large-scale pathloss channel model. To investigate the feasibility of LTE connectivity for low altitude small drones, the system performance was compared with the requirements established by 3GPP. In addition, the technical challenges of utilizing the current industrial 4G technology for providing BVLoS operations for drones were revealed. Finally, several future works and potential solutions were presented to overcome the challenges and improve the LTE connectivity performance.
- The drone power consumption was studied and the main power consuming parts were revealed. The results can be used as guidance to improve energy efficiency and drone flight time for future works.

The rest of this paper is organized as follows. Section 2 presents the methodology during the drone prototype development using an LTE mobile network-based control system and all the methods used during the field test to assess network performance. Section 3 presents the channel model and large-scale pathloss characteristics of the communications channel between eNodeB and drones. Section 4 discusses the results obtained, which show how good the network performance is by comparing a few altitudes with ground level as a benchmark. This section also discusses *RSRP* and *RSRQ* values obtained that result in HO procedure, and data rates and delays experienced by the UE at different altitudes. The last part of this section discusses the power consumption by the new system and how it will affect drone performance in many aspects, such as flight time and drone speed. Section 5 highlights practical challenges of utilizing the LTE network for providing BVLoS connectivity for drones and addresses some potential solutions to overcome the challenges. Finally, Section 6 concludes the paper.

## 2. Methodology

### 2.1. Hardware Consideration

At first, a hardware survey was conducted within the available market. Table 1 presents five short-listed products that support an LTE-based control system for a custom drone. The products’ main criteria are having real-time control while sending and receiving direct application programming interface (API) telemetry data and live-feed video footage using an LTE mobile network.

Based on the market surveys, all products offer almost the same features. However, Raspberry Pi provides unique characteristics. Raspberry Pi is a compact 64-bit microprocessor that can run a wide range of programming languages such as Python, Java (Debian Soft Float), C, Node.Js, and PHP. In addition, Raspberry Pi can run any Acorn RISC Machine (ARM)-based operating system [30]. It might be useful for the future development of the Internet of Drones (IoD). On the other hand, Raspberry Pi is very affordable and cost-saving compared to the other products.

### 2.2. Software Setup

**Figure 2:**
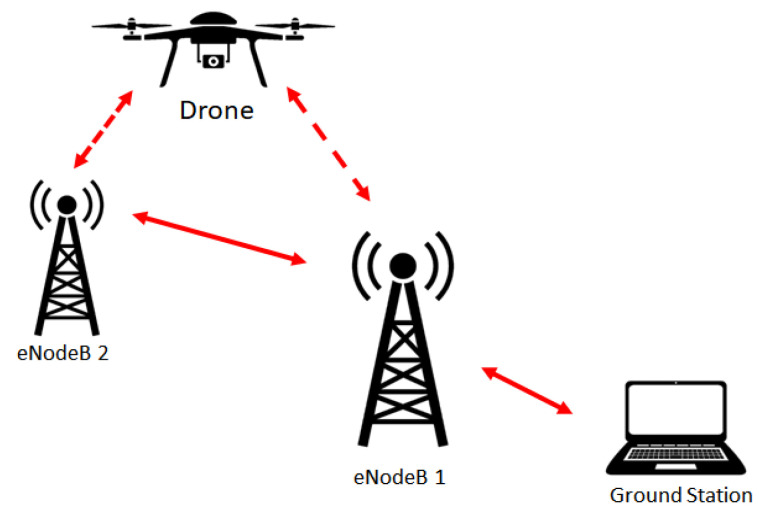
Concept of wireless communication considered.

Figure 2 depicts the general concept of wireless communications considered in this article. The ground station, which consists of a laptop or a desktop as the first UE, is equipped with Ardupilot software [36]. As the second UE, the drone is embedded with a Raspberry Pi, a Pixhawk flight controller (FC) [37], and an LTE modem. These UEs are connected to an LTE mobile network, which is conceptually connected in a single wide-area network (WAN).

**Figure 3:**
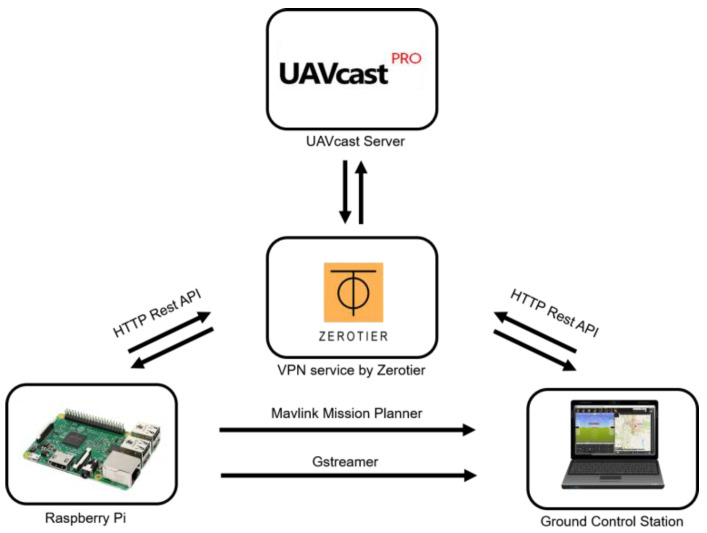
Configuration between drone and ground station with server domain.

In this article, a cloud-based operational system was implemented and a cloud server service from UAVmatrix, known as UAVcast [35], was used. As shown in Figure 3, the drone and the ground station are connected to a virtual private network (VPN) provided by Zerotier to obtain a static IP address. With some configuration, both UEs are connected to the UAVcast cloud server using LTE mobile network. The drone will send its telemetry data and live stream video to the server, then accessed by the ground station, and vice versa. Both UEs are connected via LTE mobile network.

### 2.3. Experiment Equipment

**Figure 4:**
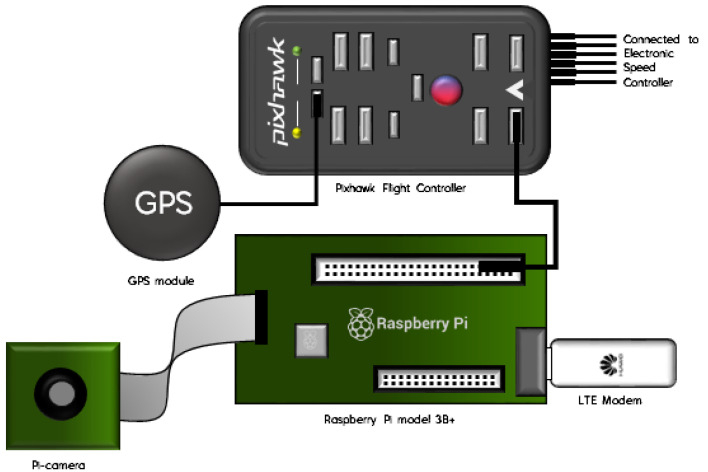
Hardware configuration for communication module of the drone prototype.

As illustrated in Figure 4, the hardware used in this research consists of a Raspberry Pi model B+, a Pi camera from Raspberry Pi, an FC from Pixhawk, a Huawei E3372 LTE modem, and a GPS module. The function of Pixhawk FC is to control the drone’s movement, assisted with a GPS module to obtain the drone’s location. CC data from the ground station is handed to FC over the Raspberry Pi, which is equipped with an active internet plan from a local network provider. To complement the standard drone operation requirement, a camera was equipped at the Raspberry Pi to stream live video footage to the ground station.

**Figure 5:**
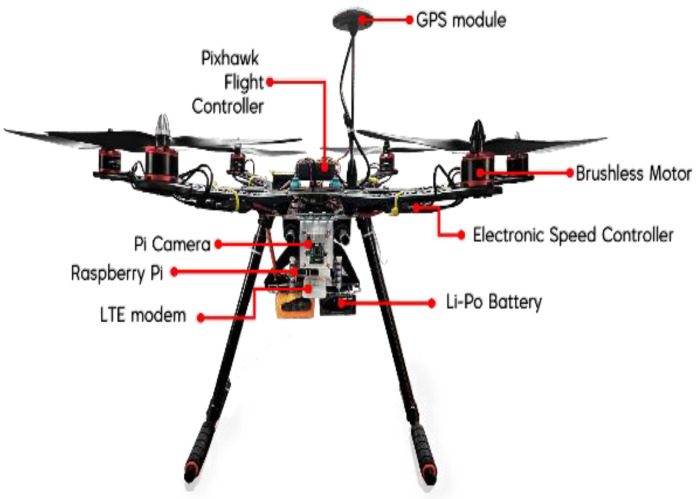
The developed drone prototype with an LTE-based control system.

Furthermore, the FC is connected to the Electronic Speed Controller (ESC), which adjoins the drone’s brushless motor. Each of the drone components is interconnected, powered by a lithium-ion polymer (Li-Po) battery. Figure 5 shows the developed LTE-powered drone prototype.

### 2.4. Measurement Area

**Figure 6:**
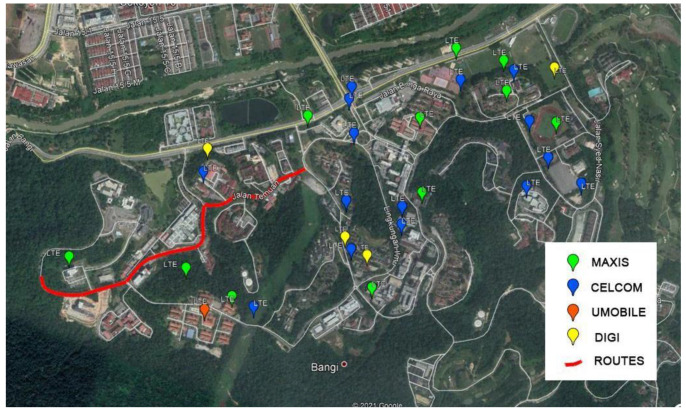
Mapped routes at the National University of Malaysia (UKM).

A 1.6 km route located at the National University of Malaysia (UKM) was chosen as the research area. The campus can be considered as a sub-urban metropolitan area with a geographic terrain of undulating hills. It is fully covered with an excellent mobile network infrastructure. The locations of available eNodeB towers were extracted from some open-based websites such as www.cellmapper.net (accessed on 20 May 2019) [38] and www.opencellid.org (accessed on 20 May 2019) [39]. The tower locations were then plotted into the map provided by Google Earth Pro software. Figure 6 shows the research area considered.

### 2.5. Comparison of Application for Drive Test

To evaluate mobile network performance at a particular location, performing a drive test is one of the preferable methods. To perform a drive test, a smartphone with an installed drive-test application is required to measure and record required data such as *RSRP*, *RSRQ*, data rates, latency, and a few other parameters.

Plenty of applications are available on Google Play Store for Android devices to perform the drive test, such as GNet Track Pro, RF Signal Tracker, Network Cell Info, and ASCOM Terms. To choose a precise application, a field test was conducted at the Kilim Geoforest Park, Langkawi, Malaysia. The location is suitable for representing the worst-case scenario for a mobile network service. Among the available applications in the market, two applications were examined for the actual evaluation: GNet Track Pro [40] and Network Cell info [41].

**Figure 7:**
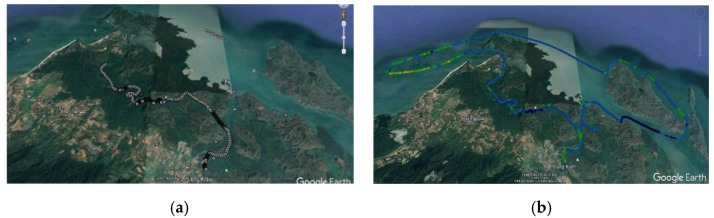
Drive test results by Network Cell Info (**a**) and GNet Track Pro (**b**).

Figure 7 depicts the drive test results, which were measured via Gnet Track Pro and Network Cell info applications. Although both applications can measure and record required data for network performance evaluation, as shown in Figure 7 there are many blank paths in the results of the Network Cell info application, which means plenty of data lost during the drive test. In contrast, the Gnet Track application did not encounter the problem, as there are no gaps generated or blank plots along the route and it can record and store data correctly. Besides, the application also supports a drive test for two cellular networks at one time. This advantage will make it easier to work for actual drive tests.

Based on this result, the G-Net Track Pro has been selected to perform a cellular network drive test for the rest of the research work. G-Net Track Pro is a tool to perform drive tests and monitor cellular network performance. This application provides a wide range of features, such as cell measurement for serving and neighbor cells in both indoor and outdoor scenarios, measurement recording, map visualization, voice, and data test sequence for uplink and downlink, and cell scanning. The authors in [42,43] utilized G-Net Track Pro to study the LTE network performance in indoor and outdoor scenarios. The results showed that G-Net Track Pro is capable of providing accurate and reliable measurement results.

### 2.6. Field Trial Measurement

**Figure 8:**
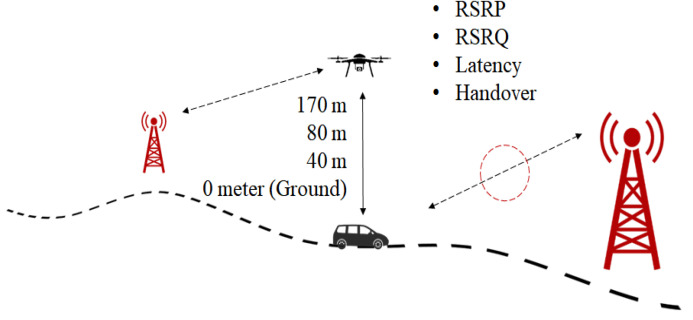
Illustration of the drive test method.

Figure 8 illustrates the concept of the drive test conducted. The measurement results were collected in a commercial LTE network with a carrier frequency of 2.6 GHz and a system bandwidth of 20 MHz. The drive test was conducted in two steps, first, at ground level and second, at higher altitudes. To do so, a Huawei STK-L22 smartphone with the preinstalled G-net Track Pro application was used. Once the application started to record, it was carried along while driving a car for the entire planned route to record the data needed to evaluate the network performance at ground level. The phone was placed inside a car without any external antenna and only powered by its own battery. Since the GNet Track Pro application’s reliability has been tested before, just a single test was conducted for each track. This test was repeated four times by using the Subscriber Identity Module (SIM) card of four different telecommunication service providers that are widely available in Malaysia: Maxis, Umobile, Celcom, and Digi. During the test, the car speed maintained at a speed around 30 km/h. The ground level measurement was performed as the benchmark. Next, higher altitude drive tests were conducted at three different altitudes: 40 m, 80 m, and 170 m on average. The height of 170 m was chosen to investigate the impact of serving drones by the sidelobes of BSs’ antennas. The intermediate height of 80 m was selected because that is close to the BS antenna height in suburban areas. Finally, the height of 40 m was considered since that is the most commonly used flying altitude for many drone applications, such as survey, inspection, and monitoring, and the drones are more likely to be served by the main lobes of BSs’ antenna. For the aerial drive test, the smartphone was attached to the developed hexacopter drone and the drone flew within the same routes as the car with an average speed of 35 km/h.

### 2.7. Feasibility Test

**Figure 9:**
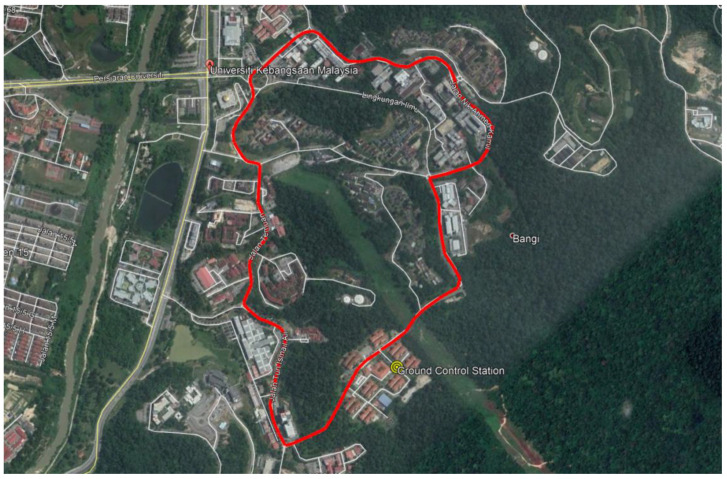
Mapped waypoint for feasibility test.

Since the network quality is still uncertain, the test was conducted at ground level. The Ground Control Station (GCS) was located at a specific place with good network quality, and the drone was brought inside a car traveling around the UKM campus. The waypoints are as shown in Figure 9. Since there is no suitable method to measure detail parameters to test the performance, this test aimed only to measure the throughput and delay experienced by the drone and the GCS. The car speed was maintained at around 45 km/h during the test. The route is around 4.2 km, and the farthest point between the drone and the GCS is about 1.2 km.

## 3. Aerial Channel Model

The propagation condition and consequently the channel characteristics between BSs and UAVs are dramatically different with BSs and terrestrial UEs; one of the main differences is the *LOS* or *NLOS* propagation conditions. In this section, we present the large-scale path loss characteristics of communications channel between BS and drones and a complete characterization of propagation channel is out of the scope of this paper.

It is highly beneficial to consider the large-scale characteristics of the propagation channel for understanding and analyzing the actual behavior of the communication link between BSs and drones. The propagation condition of *LOS* and *NLOS* can be determined based on *LOS* probability, which is a function of heights and distance of transceivers. From the 3GPP [44] the *LOS* probability, $P_{LOS}$, for aerial UEs (drones) in urban-macro scenario is given as,

(1) $$\begin{array}{cc} P_{LOS}=\{\begin{array}{lll} 1 & , & d_{2D}\leq 18m\\ \left[\frac{18}{d_{2D}}+\exp\left(-\frac{d_{2D}}{63}\right)\left(1-\frac{18}{d_{2D}}\right)\right]\left[1+C^{\prime }\left(h_{UT}\right)\frac{5}{4}{\left(\frac{d_{2D}}{100}\right)}^{3}\exp\left(-\frac{d_{2D}}{150}\right)\right] & , & d_{2D}\leq 18m \end{array} & 1.5\;m<h_{UT}\leq 22.5\;m \end{array}$$

(2) $$\begin{array}{cc} P_{LOS}=\{\begin{array}{lll} 1 & , & d_{2D}\leq d_{1}\\ \frac{d_{1}}{d_{2D}}+\exp\left(\frac{-d_{2D}}{p_{1}}\right)\left(1-\frac{d_{1}}{d_{2D}}\right) & , & d_{2D}>d_{1} \end{array} & 22.5\;m<h_{UT}\leq 100\;m \end{array}$$

(3) $$\begin{array}{cc} P_{\mathrm{LOS}}=1 & 100\;m<h_{UT}\leq 300\;m \end{array}$$

where, $d_{2D}$ is the horizontal distance between UAV and BS, $h_{UT}$ is the height of drone, and

(4) $$C^{\prime }\left(h_{UT}\right)=\;\{\begin{array}{ccc} 0 & , & h_{UT}\leq 13\;m\\ {\left(\frac{h_{UT}-13}{10}\right)}^{1.5} & , & 13\;m<h_{UT}\leq 22.5\;m\; \end{array}$$

$$p_{1}=4300\log_{10}\left(h_{UT}\right)-3800$$

$$d_{1}=\max\left(460\log_{10}\left(h_{UT}\right)-700,\;18\right)$$

According to the above equations, the $P_{LOS}$ for drone height up to 22.5 m is similar to terrestrial UEs in [45], and by increasing height to 100 the $P_{LOS}$ will be increased and for height above 100 m the $P_{LOS}\;\mathrm{is}\;\mathrm{about}\;100\%$, which means the direct ray between transceivers is clear of obstacles.

**Figure 10:**
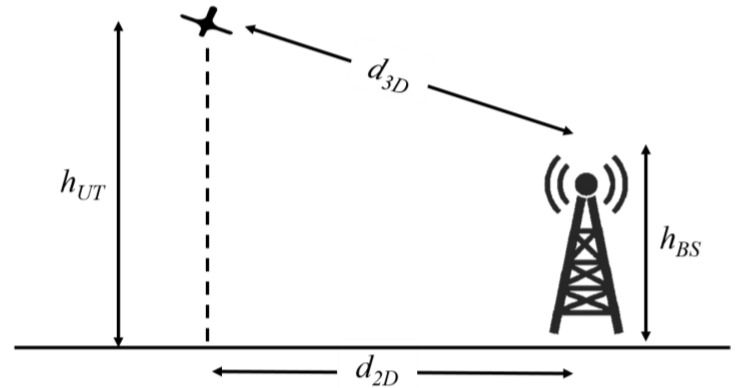
The definition of 2D and 3D distances [42].

For modeling the path loss between drone and BS, information such BS height, $h_{BS}$, drone height, 2D and 3D distances, as shown in Figure 10, are need to be known. From the 3GPP [44] the path loss model for aerial UEs is given as:

(5) $$\begin{array}{cc} PL_{LOS}=\{\begin{array}{c} PL_{1},\;10\;m\leq d_{2D}\leq d_{BP}^{’}\\ PL_{2},\;d_{BP}^{’}\leq d_{2D}\leq 5\;\mathrm{km}\; \end{array}\;PL_{1}=28+22\log_{10}\left(d_{3D}\right)+20\log_{10}\left(f_{c}\right)\;PL_{2}=28+40\log_{10}\left(d_{3D}\right)+20\log_{10}\left(f_{c}\right)\;-9\log_{10}\left({\left(d_{BP}^{’}\right)}^{2}+{\left(h_{BS}-h_{UT}\right)}^{2}\right) & 1.5\;m<h_{UT}\leq 22.5\;m \end{array}$$

(6) $$\begin{array}{cc} PL_{LOS}=28+22\log_{10}d_{3D}+20\log_{10}\left(f_{c}\right) & 22.5\;m<h_{UT}\leq 300\;m\;d_{2D}\leq 4\;\mathrm{km} \end{array}$$

(7) $$\begin{array}{cc} PL_{NLOS}=\max\left(PL_{LOS},PL_{NLOS}^{’}\right),\;for\;10\;m\leq d_{2D}\leq 5\;\mathrm{km}\;PL_{NLOS}^{’}=13.54+39.08\log_{10}\left(d_{3D}\right)+20\log_{10}\left(f_{c}\right)\;-0.6\left(h_{UT}-1.5\right) & 1.5\;m<h_{UT}\leq 22.5\;m \end{array}$$

(8) $$\begin{array}{cc} PL_{NLOS}=-17.5+\left(46-7\log_{10}\left(h_{UT}\right)\right)\log_{10}d_{3D}+20\log_{10}\left(\frac{40\pi f_{c}}{3}\right) & 10\;m<h_{UT}\leq 100\;m\;d_{2D}\leq 4\;\mathrm{km} \end{array}$$

where, $f_{c}$ is the carrier frequency, $d_{BP}^{’}$ is …, $PL_{LOS}$ is *LOS* pathloss, and $PL_{NLOS}$ is *NLOS* pathloss.

Large-scale pathloss is one of the most important factors for estimating the received signal power in a wireless system. The pathloss models in (5) and (7) are similar to terrestrial UEs in [45]. For the altitudes between 22.5 m and 100 m, where the drone is not in the *LOS* of BS, the pathloss can be calculated based on (8). For the height above 100, where the probability of *LOS* is close to 1, the propagation condition is close to free space and can be calculated by (6).

## 4. Results

This section presents the results of the drive test collected on a commercial LTE network at different heights, as well as drone energy consumption.

### 4.1. Ground Level Field Trial

To select a reliable telecommunications service provider for the drone drive test, first a ground-level drive test was conducted to evaluate the performance of four available telecommunication service providers in terms of *RSRP* and data rate. *RSRP* is a key measurement parameter indicating the received signal power level in an LTE network and can be calculated as:

(9) $$RSRP=RSSI-10\log_{10}\left(12\times N\right)$$

where, N is the number of resource blocks of the E-UTRA carrier *RSSI* measurement bandwidth and *RSSI* is the receive signal strength indicator. The *RSSI* is measured over the entire bandwidth and is the average of the total power (including intracell power, interference, and noise) observed within Orthogonal Frequency Division Multiplexing (OFDM) symbols that contain reference symbols.

**Figure 11:**
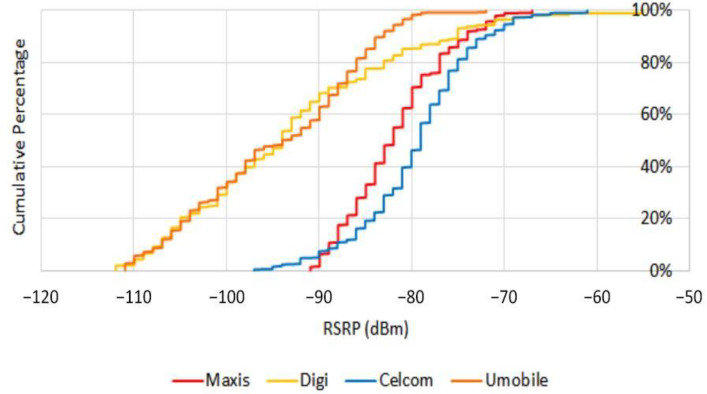
*RSRP* distribution at ground level.

Figure 11 shows the drive test results in terms of cumulative percent of *RSRP*. As results show, Maxis provides the most reliable coverage, wherein 90% of the time, *RSRP* is above −75 dBm and in 100% it lies above −90 dBm.

**Figure 12:**
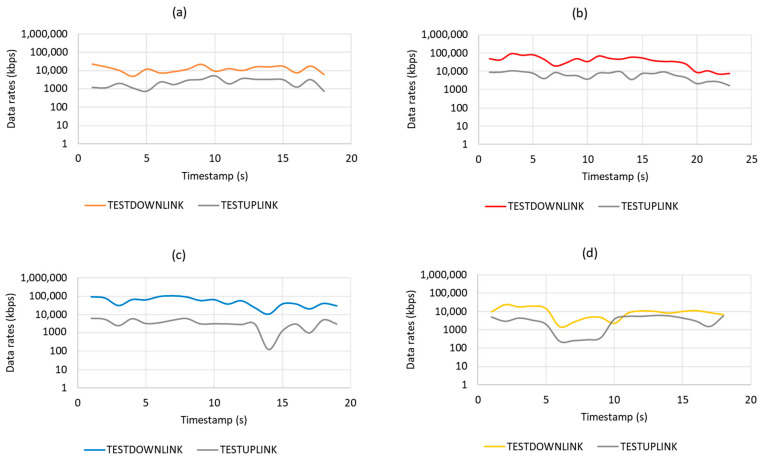
Data rates by different mobile network operator: (**a**) Umobile, (**b**) Celcom, (**c**) Maxis, and (**d**) Digi.

On the other hand, Figure 12 shows the data rate results of four service providers in both uplink and downlink. Based on the result, Maxis provides the highest throughput value with an average of 6451 kbps in uplink and 41,332 kbps in downlink. Therefore, based on the *RSRP* and uplink throughput values, the Maxis network is carried up for further evaluation.

### 4.2. Higher Elevations Field Trial

**Figure 13:**
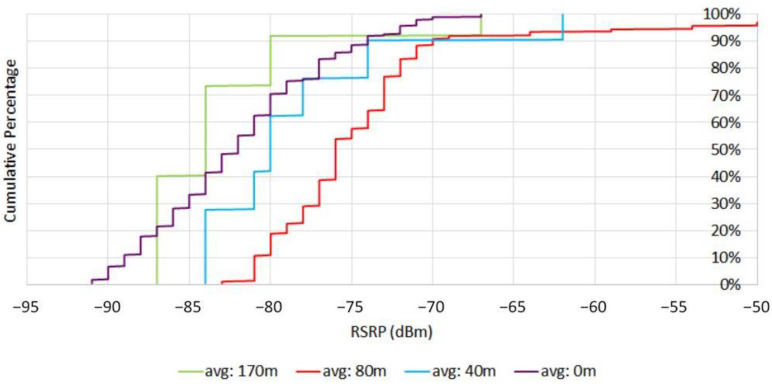
Reference signal received power (*RSRP*) distribution at different altitudes.

Figure 13 shows the cumulative distribution of *RSRP* at different elevations. By increasing the height, at first, the *RSRP* improved. According to (1)–(3), the main reason for this behavior is that by increasing height, the probability of *LOS* communications increases, resulting in lower pathloss. In addition, according to (5) and (6), when the drone altitude increases above the breakpoint pathloss is similar to the free space propagation. However, as the drone goes to higher altitudes (in this case 170 m), there is a significant loss in the *RSRP* signal level. By increasing altitude, the distance between the drone and eNodeB increases and results in larger pathloss and reduces the eNodeB antenna gain and receive power. In addition, since the eNodeB antennas are down tilted by increasing the height the probability of serving drones by mainlobes decreases. Therefore, at higher altitudes, drones will be served by the sidelobes of antennas, which results in lower antenna gain than the mainlobes of eNodeB antennas serving UEs at lower altitudes. However, the *RSRP* values are concentrated in −65 dBm and −85 dBm for different heights, which shows that coverage for drones at the measured altitudes is satisfactory.

Another key measurement parameter is *RSRQ*, which indicates the received signal quality level in a network and the effect of interference from adjacent eNodeBs. The *RSRQ* can be calculated as,

(10) $$RSRQ=\frac{N\times RSRP}{RSSI}.$$

**Figure 14:**
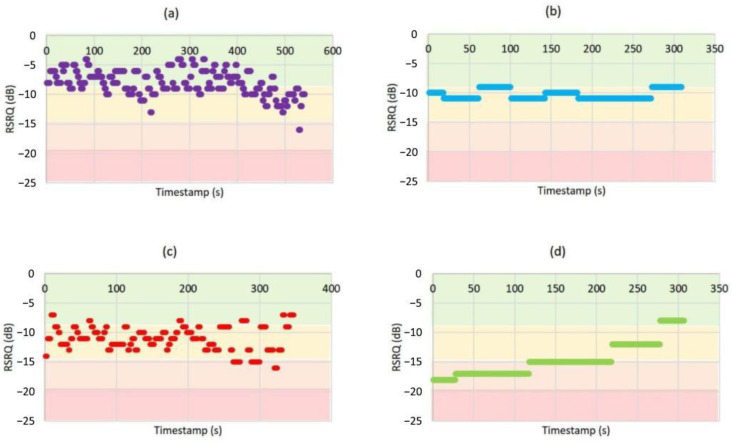
Reference signal received quality (*RSRQ*) distribution at (**a**) 0 m, (**b**) 40 m, (**c**) 80 m, (**d**) 170 m.

Figure 14 presents the *RSRQ* at different heights. As results show, by increasing the elevation the *RSRQ* level was reduced considerably. While by increasing the height the probability of LoS increases and a higher level of the desired signal can be received, but on the other hand, the interference level from adjacent cells also will be increased. It should be noted that aerial UEs can see a larger number of adjacent eNodeBs than ground UEs. Therefore, aerial UEs receive higher interference energy from the neighboring cells.

By comparing the results, it can be revealed that in higher altitudes, e.g., 170m, the *RSRQ* level decreases significantly. This is due to the ‘umbrella effect’, in which eNodeB antennas are down tilted to optimize terrestrial coverage. Besides, in such altitudes, the drone is in range of sidelobes of neighbor cells and experiences severe interference from the downlink transmissions of those cells. Therefore, in terrestrial LTE networks, drones are expected to experience more severe interference at higher altitudes than altitudes less than eNodeB height.

### 4.3. Uplink and Downlink Throughput Comparison with 3GPP Standard

The throughput values will determine the performance quality of the drone while flying using an LTE mobile network. The most important value that must meet the requirement as suggested by 3GPP in Release-15 [18] is the uplink throughput, since the drone is required to stream a live feed video from the drone to the ground station. This application will require a high bandwidth due to the size of the video itself.

3GPP command and control standards provide an essential guide to maintaining proper operational control of drones. For CC communications, a 200 kbps uplink data rate, a packet error rate of less than 0.1%, and network latency less than 50 ms is required. For payload communications, drones need to send back live telemetry data, pictures, or videos in cases such as flying cameras and remote surveillance. The drone requires around 4 Mbps data throughput for a 1080p quality streaming video resolution for the current setup.

**Figure 15:**
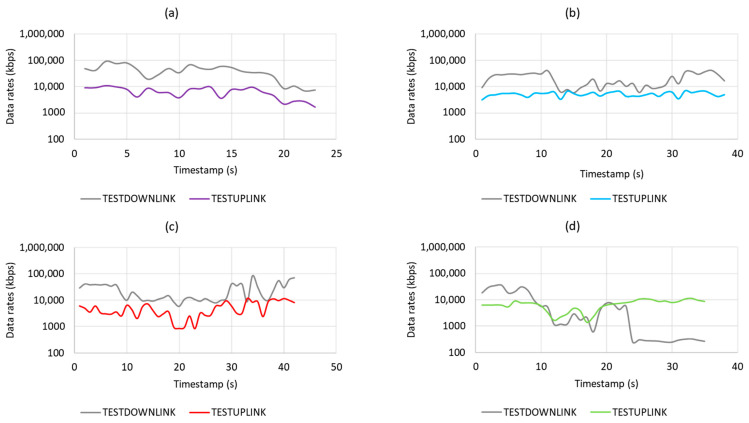
Data rates at 0m (**a**), 40m (**b**), 80m(**c**), and 170m (**d**).

Based on the field trial results, as plotted in Figure 15 and summarized in Table 2, it can be observed that the LTE network considered is capable of providing sufficient data rates with acceptable network latency for CC transmission as well as payload transmission for drone use cases. However, the results show that by increasing height, the latency increases as well, which means for use cases with the need of very low latency, e.g., remote real-time control, which needs less than 20 ms network latency, the network needs further enhancement.

### 4.4. Effect of Fly Height on Handover

One of the major technological features of the UMTS Soft/Softer handover architecture was absent when the LTE system was developed. This is mainly due to the adjacent cell frequency reuse and orthogonality in LTE, and because the LTE system has a flat architecture [46]. Therefore an assumption was made that only hard handovers occur during the test. The UE’s signal quality was analyzed to see what happened during the handoff from one eNodeB to another eNodeB along the route during the drive test.

**Figure 16:**
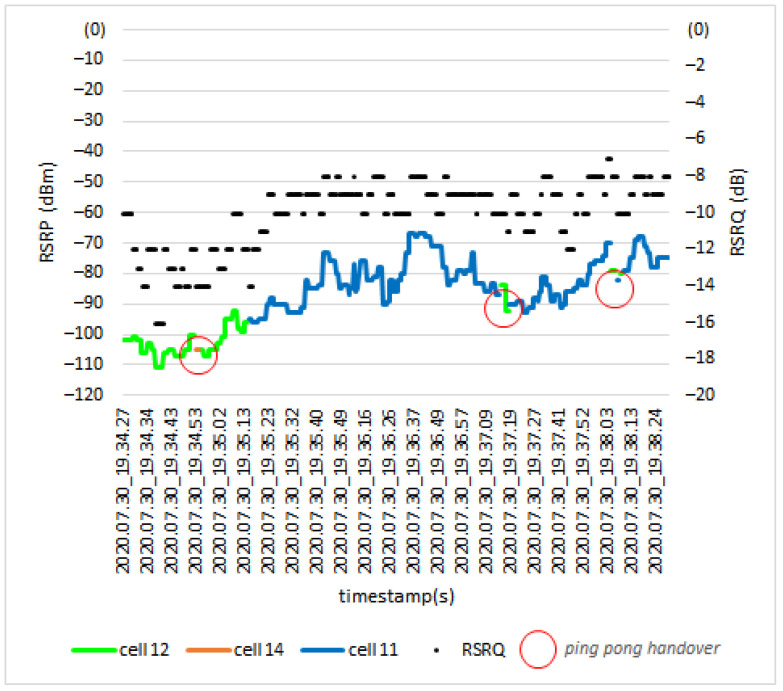
*RSRP* and *RSRQ* level during handover at 0 m (ground level).

Figure 16 shows the *RSRP* and *RSRQ* levels for a scenario where the terrestrial UE moved throughout the path. It can be seen from the results that in three points ping-pong handovers occurred (red circles). The ping-pong HO happens when a UE is handed over from one cell to another but quickly handed back to the original cell. During the drive test, the UE was served by two dominant cells: cell 12 and cell 11. When the link is handed from cell 12 to cell 11, the *RSRP* level changed from −96 dBm to −95 dBm, and the *RSRQ* level changed from −12 dB to −14 dB.

**Figure 17:**
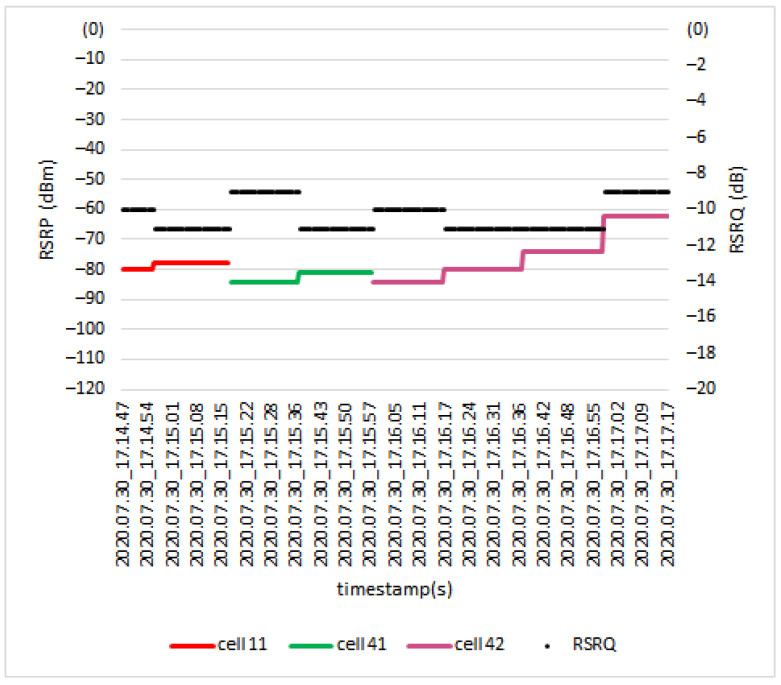
*RSRP* and *RSRQ* level during handover at 40 m.

Figure 17 shows the *RSRP* and *RSRQ* results for the drone UE when flying at the height of 40 m. Along the path, the drone was served by three different cells; Cell 11, Cell 41, and Cell 42. During the handover from Cell 11 to Cell 41, the *RSRP* and *RSRQ* values changed from −78 dBm to −84 dBm and from −11 dB to −9 dB, respectively. Next, during the handover process from Cell 41 to Cell 42, the *RSRP* value changed from −81 dBm to −84 dBm, and the *RSRQ* value changed from −11 dB to −10 dB.

**Figure 18:**
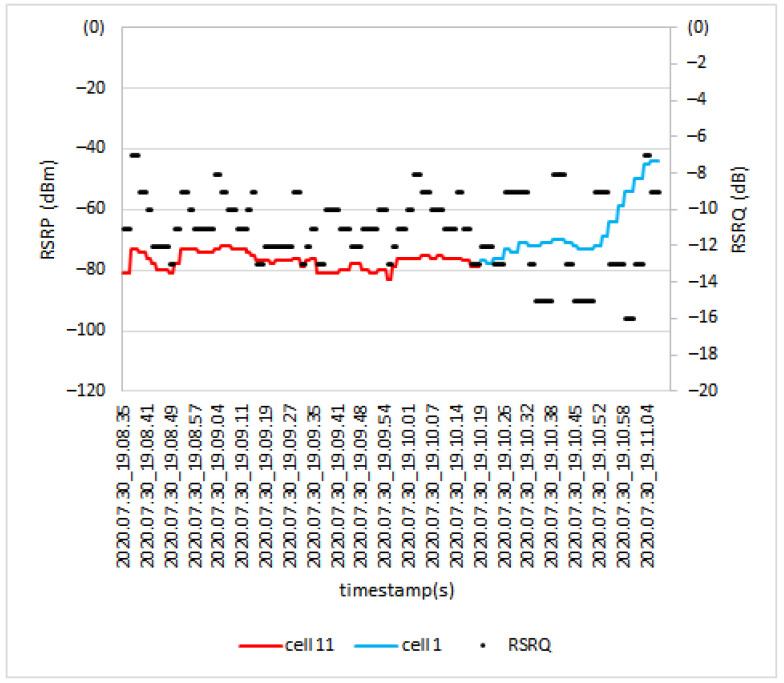
*RSRP* and *RSRQ* level during handover at 80 m.

Figure 18 shows the handover procedure at the height of 80 m. In this scenario, drone UE was served by two cells, which were Cell 1 and Cell 11. The *RSRP* value during the handover procedure from cell 11 to cell 1 changed from −79 dBm to −77 dBm and *RSRQ* changed from −13 dB to −12 dB.

**Figure 19:**
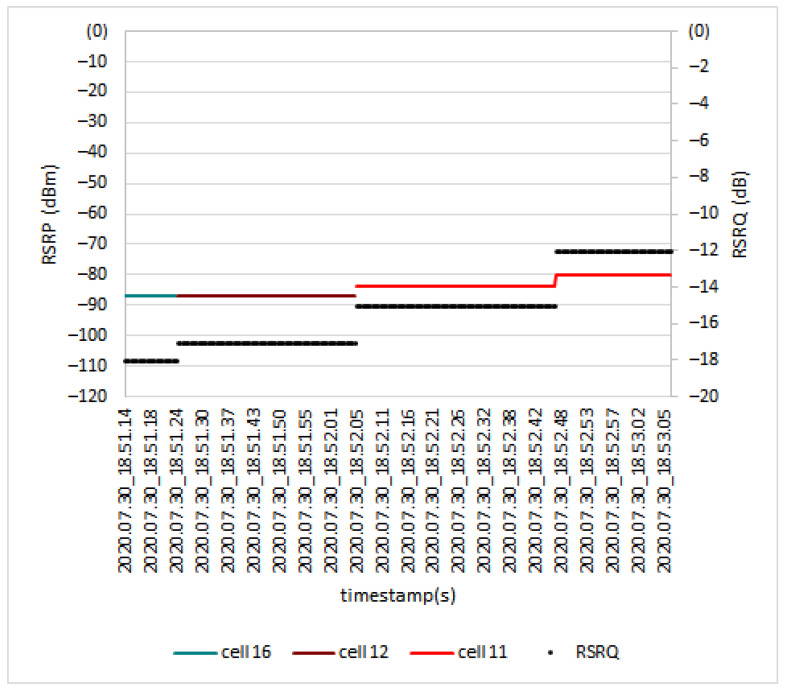
*RSRP* and *RSRQ* level during handover at 170 m.

Based on Figure 19, three dominant cells were involved in serving the UE during the test: Cell 16, Cell 12, and Cell 11. When the link was handed from Cell 16 to Cell 12, the *RSRP* level did not change, remaining at −87 dBm, and only the *RSRQ* level changed from −18 dB to −17 dB. Next, during the handover process from Cell 12 to Cell 11, the *RSRP* value changed from −87 dBm to −84 dBm, and the *RSRQ* value changed from −17 dB to −15 dB.

Based on Figure 16, Figure 17, Figure 18 and Figure 19, a conclusion can be deduced that, as the drone UE went to a higher altitude, the signal quality was slightly degraded. However, the handover still occurred in good condition because there was no glitch or loss of connection, such as a sudden drop signal during the handover.

### 4.5. Effect of Surrounding Environment on Handover

**Figure 20:**
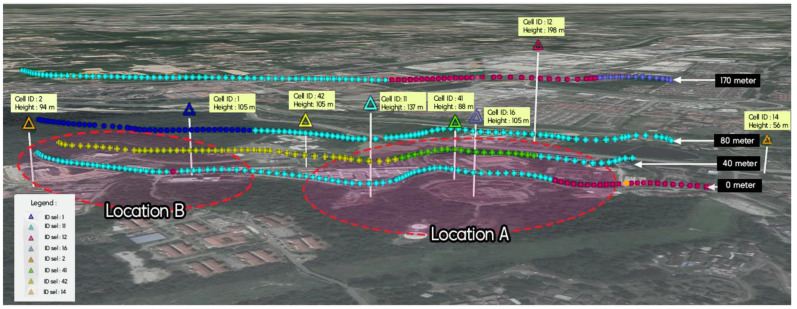
Location of user equipment (UE) and serving cell.

To provide a detailed analysis of the handover procedure at different heights, the effect of the surrounding environment on handover needs to be investigated. To do so, first, the impact of the eNodeB tower’s height on the handover decision making is considered. Table 3 presents the height of considered eNodeB towers. As shown in Figure 20, by changing the flight height, the drone is served by a different set of cells. When the drone flies at altitudes lower than eNodeB height, it is usually served by the nearby eNodeBs. This is because the eNodeB antennas are downtilted to optimally serve terrestrial UEs. Therefore, drone UEs will be served by the main lobes of eNodeB antennas. While for cases in which the drone flies beyond the height of eNodeB towers, the drone is served by sidelobes of the antennas. As can be seen from the results of the 170 m height route, the drone UE was served by Cell 12, which is located farther than other cells in this area. That means the signal strength of Cell 12 sidelobes is stronger than sidelobes of adjacent cells, or the route is located at the null of adjacent cells sidelobes.

To investigate the effect of geographic terrain and the surrounding environment, the research area was narrowed into two locations, Location A and Location B, as shown in Figure 20. By comparing the results of heights 0 m and 40 m, it can be revealed that when the drone flies above the height of buildings and trees, where the probability of LoS is higher than ground UEs, the drone is almost served by nearby eNodeBs, while for terrestrial UEs scenario of serving cell selection impacts by factors such as signal blocking and shadowing effects from the trees and buildings.

### 4.6. Drone Performance Evaluation

**Figure 21:**
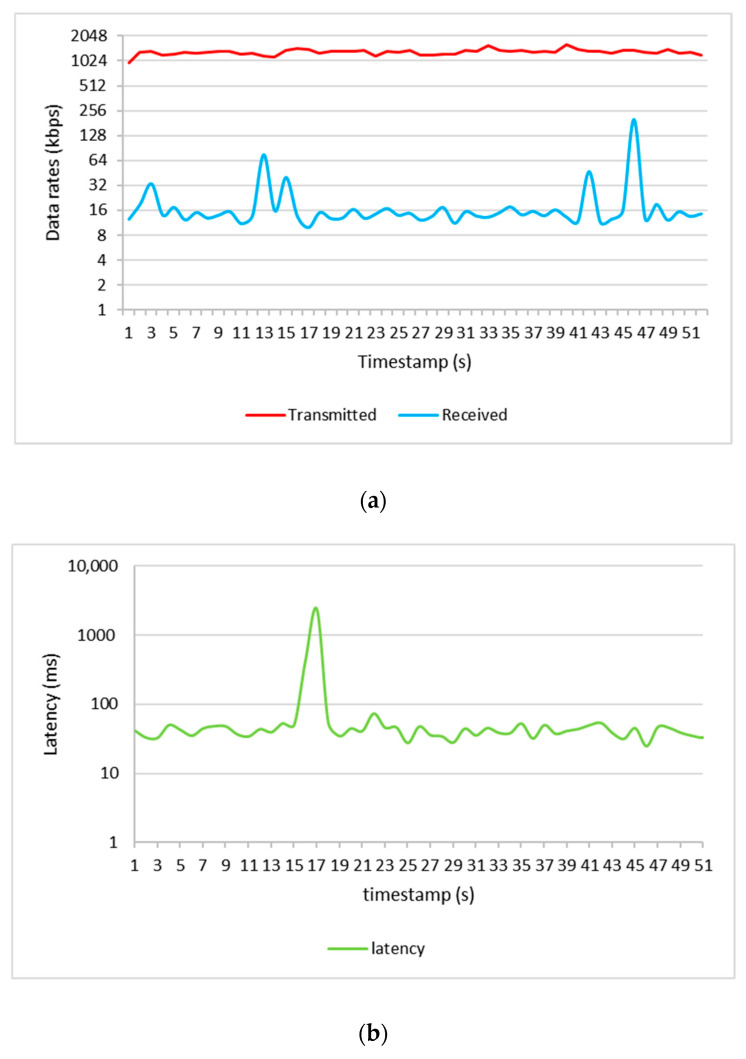
Packets data transmitted and received by the drone (**a**) and its delays (**b**).

Figure 21 shows the results of the data rate as well as latency for packet transmission from the drone to the ground station and vice versa. The average packet data transmitted from the drone to the ground station was 1303 kbps, and the average data received was 20 kbps with a delay of 94 ms for both uplink and downlink. However, according to the 3GPP release-15 standard [19], the data rate requirement for CC and payload communications in uplink is 100 kbps and 4 Mbps with a latency of less than 50 ms. Therefore, the performance of the current LTE network needs to be improved for providing connectivity for BVLoS drone use cases that require real-time control and high-quality image or video transmission.

### 4.7. Energy Consumption

The motors used by the drone are DJI 920kV motors with a size of 22 mm × 12 mm and 1045 propeller. Table 4 presents the details of energy consumed by the drone in the different throttle levels. The battery utilized is a 3-cell Li-po battery with an output of a maximum of 12.6 V and has a capacity of 6000 mAh with a discharge rate of 60 C.

To determine the flight time, *T*, of the drone, the total current, $I_{t}$, needs to be calculated. In this regard, first, the total current consumed by the motors, $I_{tm}$, should be obtained [41].

(11) $$I_{tm}=N\times I,$$

where *N* is the number of motors, and *I* is the current for a single motor.

After the total current of the motors is obtained, the total current consumed by the drone can be obtained by adding it up with the total current consumed by every component on the drone, $I_{s}$ as in Table 5:

(12) $$I_{t}=\;I_{tm}+\sum^{\;}I_{s}.$$

Thus, the flight time is the summation of battery capacity, *C*, divide by the total current consumed:

(13) $$T=\;\sum^{\;}\frac{C}{I_{t}}\times 1000.$$

**Figure 22:**
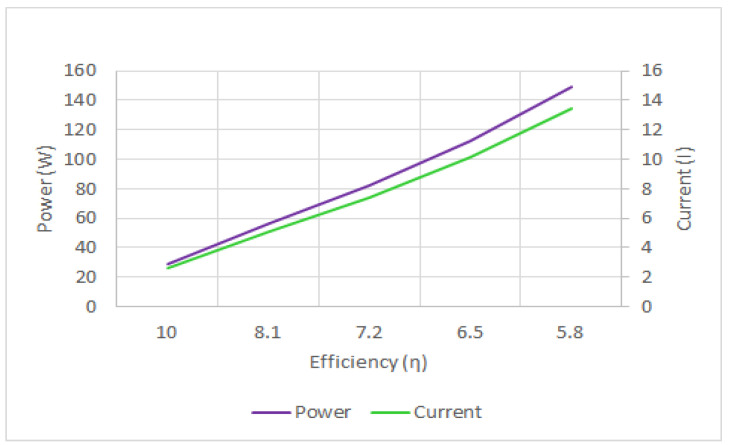
Power and current consumption versus efficiency.

**Figure 23:**
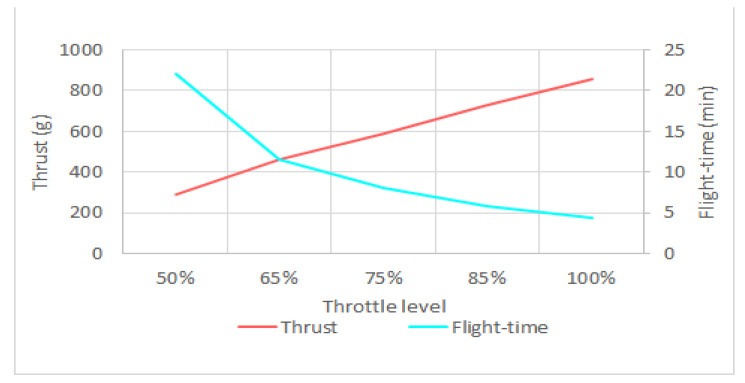
Relationship between throttle level, thrust, and flight-time.

As can be seen in Figure 22, the drone’s power and current consumption are inversely related to efficiency. In other words, by further pushing the throttle, the rate of power consumption enhancement is more than the thrust enhancement and a larger part of the energy will be lost at higher throttle levels. Hence, as the throttle is pushed further, the drone’s flight time will decrease, as shown in Figure 23. Moreover, the results show that the energy consumption by control and telecommunications components is relatively high, compared to the energy consumed by the motors. Therefore, to enhance the drone’s flight time, not only should the drone weight, battery capacity, and throttle level be optimized, but also more energy-efficient components and algorithms should be developed for communications and flight control.

## 5. Reflection: Practical Challenges and Solutions

Existing LTE infrastructure mainly focussed on terrestrial communication. To make sure the area has LTE network coverage, a pre-flight setup needs to be conducted, by using an radio-controlled drone and doing a drive test first. The feasibility of using the sidelobe of the antenna in handling the UAV is still uncertain. The drone in this test was still flying within a coverage which theoretically looks like an umbrella provided by the eNodeB. The drone was not able to fly higher than that. This paper aimed to reveal the limitations of the current LTE design, and thus to fully optimize LTE-based control systems we need to incorporate LTE tower designers. However, this study shows that the utilization of the LTE network for drone communication is still possible. Table 6 compares the requirements made by 3GPP release-15 and the measured network performance based on the field test.

Based on the field test, the LTE-powered drone developed is able to transfer all of the telemetry data, update its waypoint, and perform real-time piloting by utilizing the Transmission Control Protocol (TCP) link at a 115,200 baud rate. Meanwhile the real-time video was streamed simultaneously by utilizing the User Datagram Protocol (UDP) link at a 57,600 baud rate. Therefore, in terms of practicality, the LTE-based control system can compensate for the drone operational requirements.

However, in QoS, the drone experienced a longer delay, which was almost twice the delay proposed by 3GPP. In addition to evaluating the QoS, the throughput used for telemetry data transmission, for both uplink and downlink, is sufficient, but for video streaming requirements it still cannot abide by the standard established by 3GPP.

In terms of practicality, the current network can provide all of the requirements needed for a drone to be operated using an LTE network, but in terms of QoS, the LTE network requires more improvements.

In addition, in terms of improvement from the conventional drone, using an LTE-based control system gives more benefits, as shown in Table 7, which allows real-time GPS tracking, the ability to update navigation data, and data transmission from sensors located at the drone. Moreover, the most important improvement is being able to do a BVLoS flight operation.

In order to enhance the performance of LTE connectivity for drones, some technical challenges, such as down-tilted BS antennas and inter-cell interference, need to be addressed.

Interference is one of the inherited issues of cellular networks which has been widely studied, and a rich set of advanced interference mitigation solutions have been developed especially for LTE networks overthe last decade [47]. Drones generate more uplink interference to neighbor cells while experiencing more downlink interference from those cells. To prevent performance degradation, the interference needs to be appropriately controlled and managed.

Some of the prominent interference mitigation techniques are Coordinate Multi-Point (CoMP) [48], interference rejection combining, and network-assisted interference cancelation and suppression [49]. However, both techniques are based on adjacent cells’ cooperation, which results in high overhead on the interface between adjacent cells (known as X2). Another strategy that can be used is radio resource partitioning, in which the assigned resources for aerial connectivity need to be orthogonal with utilized resources for terrestrial communications. However, in using such techniques efficiency of resource partitioning should be taken into account. The power control technique is another effective technique that can be used to mitigate interference, especially in the uplink.

Beamforming is another technique that is supported in LTE. In this technique, the antenna beams are concentrated in a specific direction and thus increases the reach by separating users in space. This technique can be considered an effective solution since drones are in LoS of a large number of BSs, in which omni directional antennas will cause severe interference in the downlink to drones [50]. To address the challenge of a down-tilted BS antenna, one solution is the use of dedicated cells for UAV connectivity where the antenna is pointed towards the sky. Thus, the use of dedicated directional antennas and radio resources for providing drone connectivity can be extremely useful in enhancing aerial coverage and LTE connectivity performance and suppressing the interference in both uplink and downlink channels.

## 6. Conclusions

This study aimed to reveal the potentials and limitations of commercial LTE networks for providing cellular connectivity for BVLoS drone operations. In this regard, first, an LTE-powered drone prototype was constructed. The drone is capable of extracting the potential of the current LTE network and provide a packet data rate of 1303 kbps from the drone to the ground station and a packet data rate of 20 kbps from the ground station to the drone with a delay of 94 ms for both uplink and downlink. The results show that for more advanced drone use cases where low latency and high uplink data rate are required, the current 4G network needs to be improved. Furthermore, a comprehensive field trial measurement was conducted to reveal the current cellular network’s capability and challenges. The results showed that although the current 4G network is capable of providing command and control, as well as a payload communications link for the drone BVLoS use cases, further enhancements are needed to provide a secure and reliable communications link for advanced use cases of drones, especially in terms of overall network latency and uplink data rate.

## Figures and Tables

**Table 1:** Comparison of hardware available in the market. API, application programming interface; BVLoS, beyond the visual line-of-sight;LTE, Long Term Evolution; OTP, one time purchase.

	Product Available on the Market				
	XB Station [31]	Skydrone FPV [32]	Botlink [33]	Flytpi [34]	Raspberry pi [30]
Diagram	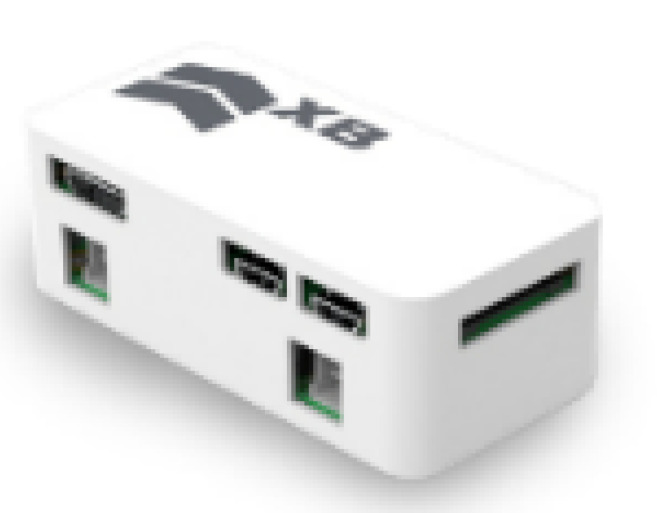	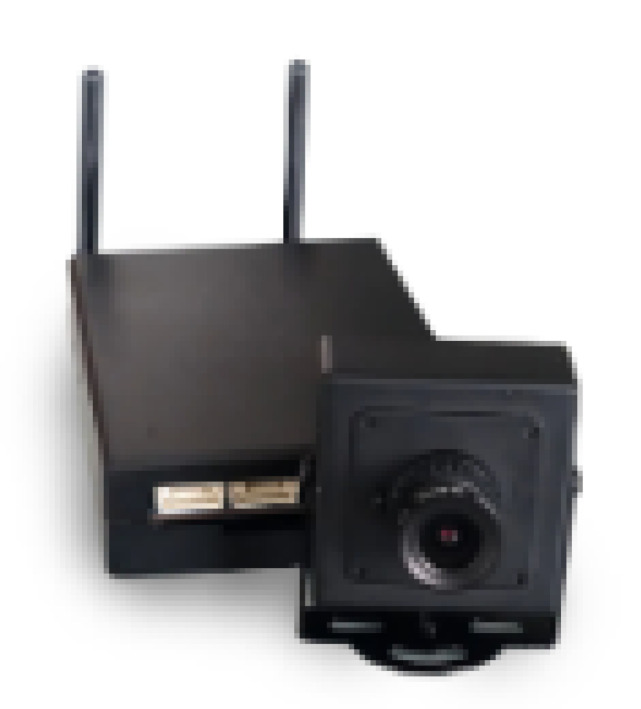	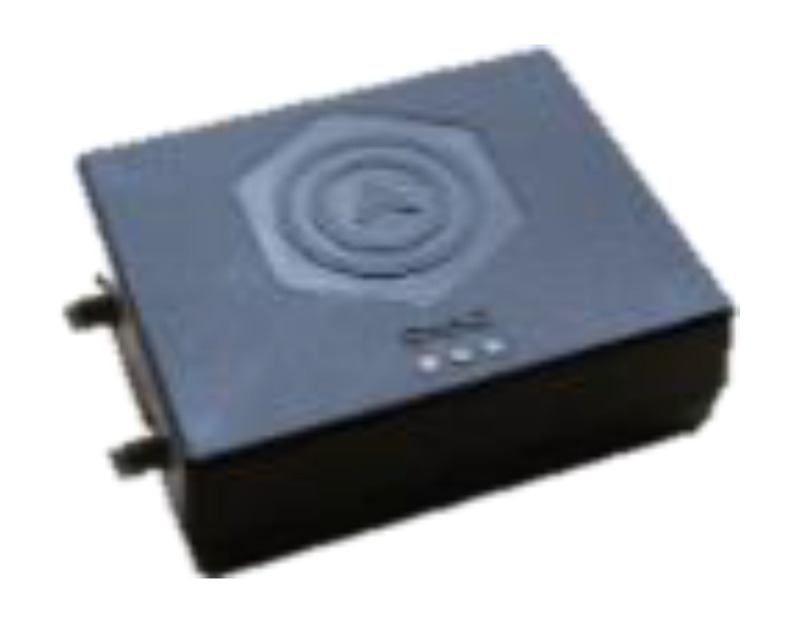	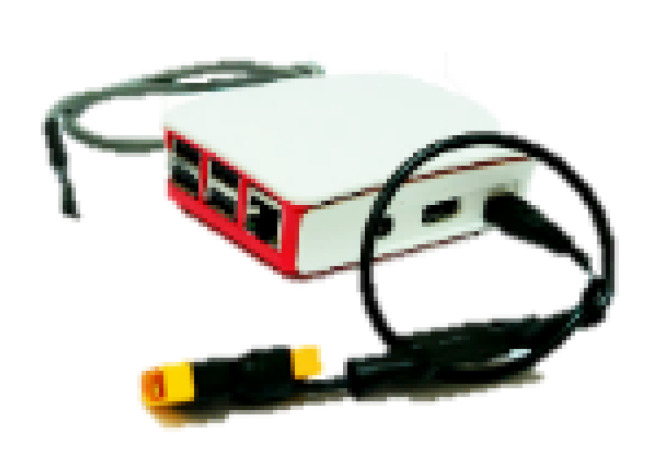	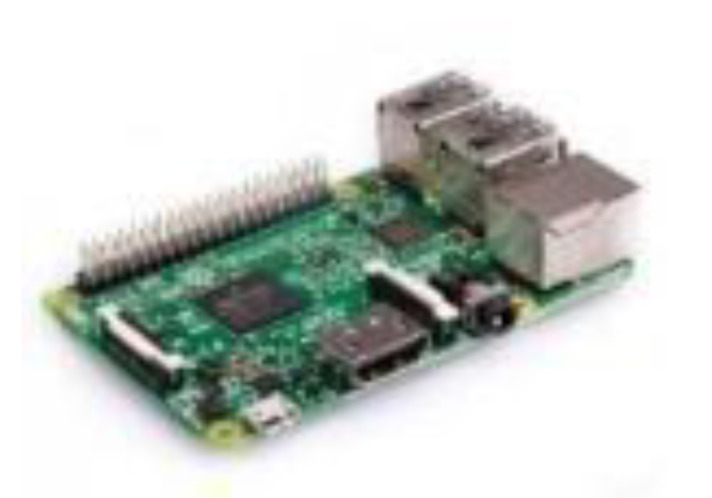
Description	Real-time control with live-feed footage using 4G network Direct API telemetry data connection BVLoS drone control	Real-time control with live-feed footage using 4G network Direct API telemetry data connection BVLoS drone control Camera gimbal control Special software for drone users	Real-time control with live-feed footage using 4G network Direct API telemetry data connection BVLoS drone control	Real-time control with live-feed footage using 4G network Direct API telemetry data connection BVLoS drone control Direct plug and play concept Special software for drone users	Aimed for Real-time control with live-feed footage using 4G network Direct API telemetry data connection BVLoS drone control
Hardware Requirement	LTE modem Flight Controller Drone Camera Battery	Flight Controller Drone Battery	Flight Controller Drone Camera Battery	LTE modem Flight Controller Drone Camera Battery	LTE modem Flight Controller Drone Camera Battery
Software	XB Firm	Sky Drone FPV	Botlink Relay	Flyt OS	UAVcast-Pro [35]
Price	$430 OTP $20/month	$999OTP	$1400 OTP $10/month	$399 OTP $499/year	$39 OTP $59/year

**Table 2:** Average throughput and delay.

Elevation	0 m	40 m	80 m	170 m
Average Uplink Throughput (kbps)	6451	5245	5051	6737
Average Downlink Throughput (kbps)	41,332	20,164	24,699	7893
Average delay (ms)	25.4	24.73	23.44	36.63

**Table 3:** Cell ID and its height.

Cell ID	Height (m)
1	105
2	94
11	137
12	198
14	56
16	105
42	105
41	88

**Table 4:** Specification for the energy consumption of the drone.

Throttle	Current (A)	Power (W)	Thrust (g)	Efficiency (g/W)	Temperature (°C)
50%	2.6	28.9	290	10.0	55 °C
65%	5.1	56.6	460	8.1	
75%	7.4	82.1	590	7.2	
85%	10.1	112.1	730	6.5	
100%	13.4	148.7	860	5.8	

**Table 5:** Current consumption on every component.

Component	Current (I)	Power (W)	Percentage
Raspberry Pi	360 mA	1.8 W	49.29%
Pi Camera	90 mA	0.45 W	12.32%
LTE Modem	290 mA	1.45 W	38.34%
GPS Module	0.06 mA	0.003 W	0.01%
Pixhawk FC	0.25 mA	0.0125 W	0.03%
**Total**	**730.31mA**	**3.7155 W**	**100%**

**Table 6:** Comparison between the requirement established in 3rd Generation Partnership Project (3GPP) Release-15 and the measured network performance. TCP, Transmission Control Protocol; UDP, User Datagram Protocol.

	**3GPP Standard**		**Performance Based on Field Test**	
	Command and Control	Application Data	TCP	UDP
Data type example	Telemetry data Waypoint update for autonomous aerial vehicle operation Real-time piloting Navigation database update	Video streaming Image transfer Transmission for other sensor data	Telemetry data Waypoint update for autonomous aerial vehicle operation Real-time piloting Navigation database update	Video streaming Image transfer
Latency	One way radio interface latency of 50ms from eNodeB to aerial UE	Similar to LTE terrestrial UE’s	94 ms	
Uplink/downlink data rate	60–100 kbps for both uplink and downlink	Up to 50 Mbps for uplink	Uplink: 1303 kbps Downlink: 20 kbps	

**Table 7:** Comparison between the conventional and the developed LTE-powered drone.

	Conventional Drone	LTE-Powered Drone
Data type	Telemetry data Autonomous waypoints update Real-time drone control Video streaming Image feed	Telemetry data Autonomous waypoints update Real-time drone control Video streaming Image feed Real time GPS tracking Navigation database update Transmission for other sensor data
Frequency	Telemetry: ~2.4 GHz Video: ~5.0 GHz	4G LTE~2.6 GHz
Flight distance	Line of sight	Beyond the line of sight

## Data Availability

The data presented in this study are available on request from the corresponding author. The data will be made publicly available once the drive test measurement campaign has been completed based on various drone deployment scenarios.
